# The impact of artificial intelligence on periodontal disease detection and treatment

**DOI:** 10.3389/fdmed.2026.1784123

**Published:** 2026-02-06

**Authors:** Bianca Maria Messina, Alessandro Polizzi, Angela Angjelova, Elena Jovanova, Gianluca Tartaglia, Gaetano Isola

**Affiliations:** 1 Unit of Periodontology, Department of General Surgery and Medical-Surgical Specialties, University of Catania, Catania, Italy; 2 International Research Center on Periodontal and Systemic Health “PerioHealth”, University of Catania, Catania, Italy; 3 Fondazione IRCCS Cà Granda Ospedale Maggiore Policlinico, Milan, Italy; 4 Department of Biomedical, Surgical, and Dental Sciences, University of Milan, Milan, Italy

**Keywords:** AI-based diagnosis and treatment, artificial intelligence, chatbots in dentistry, machine learning in periodontology, periodontal disease, periodontitis

## Abstract

Periodontal disease (PD) is one of the most prevalent chronic inflammatory non-comunicable diseases worldwide. Early diagnosis and timely intervention for periodontitis are essential to prevent the onset and progression of the disease, especially due to the associated risk, sometimes subclinical, of negative correlations with various systemic diseases that impair quality of life. In this regard, artificial intelligence (AI) has emerged as a transformative tool in healthcare, and its application in the detection and treatment of periodontal disease holds considerable promise. This study aims to review and update the latest evidence on the role of AI in the diagnosis and management of periodontal disease, emphasising advancements in machine learning (ML) algorithms, diagnostic imaging, and predictive modelling. Moreover, it is analyzed how AI-driven technologies, such as deep learning models applied to radiographs and clinical data, can enhance diagnostic accuracy, predict disease progression, and assist in personalized treatment planning. The potential of AI to optimise clinical workflows and improve patient outcomes is also discussed, alongside the challenges of integrating it into routine dental practice, including ethical considerations and data privacy concerns. This review highlights the current state of AI in periodontology, identifies key research gaps, and offers recommendations for future directions in AI-driven periodontal care.

## 1 Introduction

**Figure 1:**
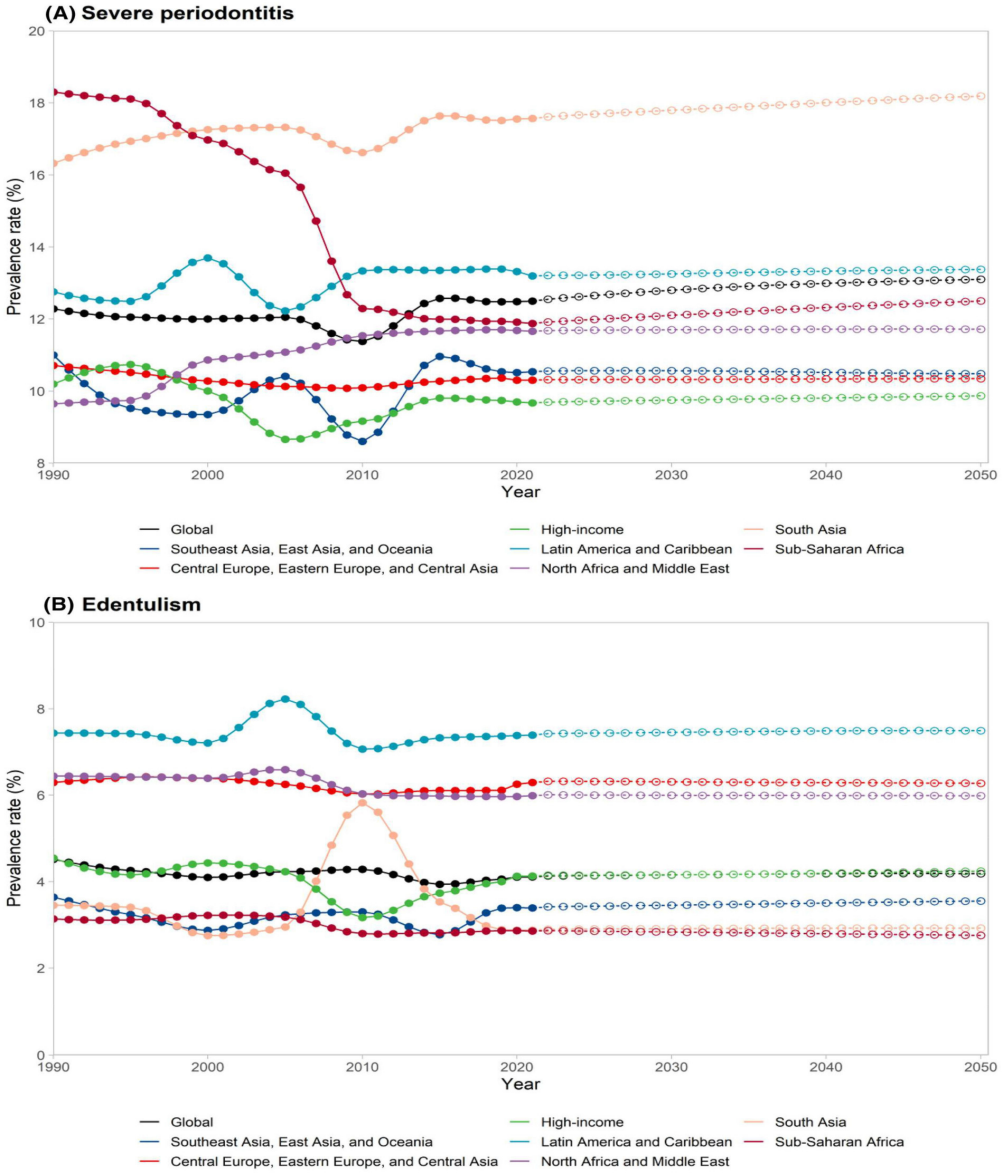
Prevalence of **(A)** severe periodontitis and **(B)** edentulism by GBD super region from 1990 to 2050. Reproduced from Nascimento et al. (22), under the terms of the Creative Commons CC BY license.

Periodontal disease (PD) is one of the most common chronic inflammatory conditions worldwide, representing a major cause of tooth loss and contributing substantially to the global burden of oral diseases (Figure 1). Epidemiological studies indicate that periodontal diseases affect a large proportion of the adult population globally, with prevalence estimates varying widely depending on diagnostic criteria, disease definitions, and population characteristics (1). Beyond its local impact, PD has been increasingly linked to systemic diseases such as diabetes, cardiovascular disorders, and certain cancers. Early detection and timely intervention are therefore crucial to prevent disease progression and its systemic complications (2). However, traditional diagnostic approaches, based on clinical probing, radiographic evaluation, and visual assessment, are often limited by subjectivity, operator variability, and their inability to detect subtle early-stage changes (3).

In recent years, artificial intelligence (AI) has emerged as a transformative force in healthcare, capable of analyzing large, complex datasets with speed and precision. In dentistry, particularly in periodontology, AI-based technologies such as machine learning (ML) and deep learning (DL) offer new opportunities to enhance diagnostic accuracy, risk assessment, and treatment planning (4). By integrating radiographic, clinical, and even microbiological data, AI systems can assist clinicians in recognizing subtle disease patterns that may go unnoticed with conventional diagnostic methods (5). Recent developments have further expanded this potential, with deep learning models now capable of analyzing standard intraoral photographs to screen for signs of periodontitis. Tao et al. demonstrated that AI-based photo processing can effectively identify periodontal inflammation and bone loss, underscoring the promise of image-based deep learning for rapid, non-invasive, and scalable screening approaches, including tele-dentistry applications (6).

Despite the rapid growth of artificial intelligence applications in periodontology, there is currently no formal consensus or universally accepted guidelines on the clinical implementation, validation standards, or ethical governance of AI-based tools for periodontal diagnosis and treatment. Most available AI systems remain investigational, being characterized by heterogeneous study designs, limited external validation, and unclear pathways for integration into routine periodontal practice (7).

In this context, this review aims to provide a concise, up-to-date overview of current applications of artificial intelligence in the detection and treatment of periodontal disease. It highlights how AI-driven diagnostic tools, predictive models, and patient-centred digital systems can optimise clinical workflows and improve treatment outcomes. Moreover, the review discusses current limitations, ethical challenges, and future research perspectives required to integrate AI safely and effectively into periodontal practice.

## 2 Materials and methods

### 2.1 Inclusion and exclusion criteria

This review was conducted through an electronic search of the scientific literature to identify relevant articles addressing the application of artificial intelligence in the diagnosis, prognosis, and treatment of periodontal disease. The PubMed/MEDLINE database was searched using combinations of keywords related to artificial intelligence, machine learning, deep learning, and periodontal disease.

Articles addressing AI-based diagnostic, prognostic, or therapeutic applications with potential clinical relevance in periodontology were included. Articles focusing exclusively on the development of technical algorithms without clinical applicability, studies unrelated to periodontal outcomes, and reports lacking sufficient methodological or clinical information were excluded.

### 2.2 Applications of AI in periodontal diagnosis and treatment

Artificial intelligence (AI) and machine learning (ML) technologies, particularly deep learning (DL), are increasingly influencing periodontal diagnostics and clinical decision-making. Traditional diagnostic approaches, such as probing depth measurements and two-dimensional radiographic assessment, are inherently limited by examiner-dependent variability, measurement error, and reduced sensitivity in detecting early or subclinical periodontal changes, especially in initial disease stages (5).

In this context, AI-driven systems based on ML and DL architectures offer the ability to process large volumes of heterogeneous data, including clinical parameters, radiographic images, and, in selected research settings, microbiological or omics-related information. By learning hierarchical representations directly from imaging and structured datasets, these models enable automated feature extraction and pattern recognition that may support more objective and reproducible periodontal assessment (8).

Rather than replacing clinical judgment, AI-based tools are increasingly being developed as decision-support systems, with the potential to assist clinicians in disease detection, risk stratification, and outcome prediction. However, the clinical performance and generalizability of these systems remain highly dependent on data quality, model validation strategies, and the clinical relevance of selected endpoints, underscoring the need for cautious interpretation of reported accuracy metrics (9, 10).

### 2.3 Diagnostic applications

A major area of AI implementation in periodontology concerns the automated detection and assessment of periodontal disease using imaging data. Among the different diagnostic tasks, the identification and quantification of alveolar bone loss on dental radiographs represents the most extensively investigated application to date. Deep learning (DL) models, particularly convolutional neural networks (CNNs), have been trained on periapical, bitewing, and panoramic radiographs to detect periodontal bone loss, classify disease severity, and support staging procedures (11).

Several studies have reported diagnostic performances comparable to those of experienced clinicians under controlled conditions. Chen et al. developed a hybrid DL framework combining YOLOv8, Mask R-CNN, and TransUNet to localise alveolar bone loss and classify periodontal disease stages on panoramic radiographs, achieving classification accuracies exceeding 90% otheir test dataset (12). However, these results are largely derived from single-center, retrospective datasets, and their generalizability to different imaging devices and clinical settings remains uncertain.

Evidence from secondary research supports the potential of AI-based radiographic diagnostics, while also highlighting relevant limitations. A recent systematic review and meta-analysis reported pooled sensitivity and specificity values of approximately 87% and 76%, respectively, for AI-assisted detection of periodontal bone loss on radiographs (11). Notably, substantial heterogeneity was observed across studies, reflecting differences in reference standards, disease definitions, and validation strategies.

Beyond radiographic imaging, AI-based diagnostic approaches have expanded to include intraoral photography. Deep learning models trained on standard intraoral photographs have demonstrated the ability to identify signs of gingival inflammation and periodontal disease without the need for radiographic input, offering a non-invasive and low-cost screening alternative. Tao et al. showed that photo-based DL systems could detect periodontitis-related features with promising accuracy, supporting their potential use in large-scale screening and tele-dentistry contexts (6). Similarly, a systematic review by Patel et al. reported wide-ranging diagnostic accuracies for image-based AI systems, largely influenced by image quality, dataset size, and annotation methods (13).

**Figure 2:**
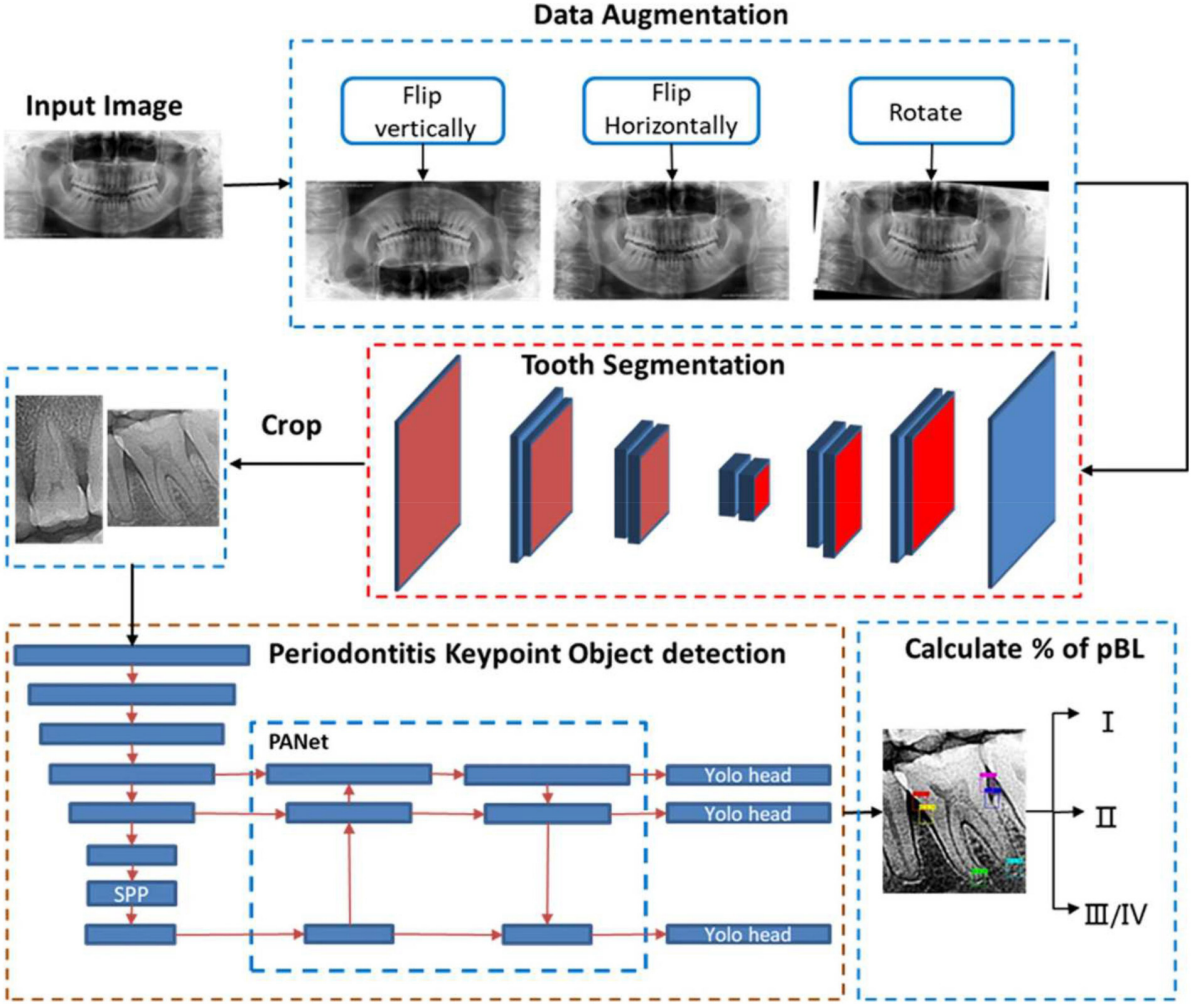
Workflow of the model training. Reproduced from Jiang et al. (23), under Creative Commons Attribution 4.0 International License.

AI applications in periodontal diagnosis are not limited to imaging alone. Supervised machine learning algorithms have been applied to structured clinical data, integrating parameters such as probing depth, clinical attachment loss, bleeding on probing, and patient-related factors to support disease classification and early risk identification (5). While these models show promise for augmenting diagnostic decision-making, their clinical utility depends on robust validation and careful alignment with established periodontal diagnostic frameworks using data augmentation (Figure 2).

### 2.4 Therapeutic and prognostic applications

Artificial intelligence has been increasingly explored as a support tool for clinical decision-making and prognostic assessment in periodontology. Unlike diagnostic applications, therapeutic uses of AI are predominantly focused on outcome prediction and risk stratification rather than direct treatment recommendation. Predictive models trained on longitudinal clinical datasets have been developed to forecast treatment outcomes, such as probing pocket depth reduction or clinical attachment level gain following non-surgical periodontal therapy (14). These models have also been applied to predict risk of disease progression and to support decision-making regarding surgical interventions when indicated.

These prognostic models integrate patient-related variables, baseline periodontal parameters, and behavioral risk factors to estimate treatment response and disease progression over time, thereby supporting individualized treatment planning. By combining these clinical parameters, AI can help clinicians identify high-risk patients, prioritize preventive or intensive interventions, and optimize treatment sequencing. In their narrative review, Patel et al. highlighted the potential role of AI-based decision-support systems in optimizing treatment sequencing, identifying high-risk patients, and improving prognostic assessment, while emphasizing that such tools should complement clinical expertise (13).

Beyond conventional outcome prediction, recent studies have explored AI models capable of stratifying patients according to expected therapeutic benefit, recurrence risk, and maintenance needs. Machine learning algorithms integrating systemic conditions, smoking status, and prior treatment response have shown potential in identifying patients more likely to experience residual pockets or disease progression despite standard therapy, thereby supporting personalized maintenance strategies (8, 15).

Beyond outcome prediction, AI-driven digital health tools are emerging as adjuncts for patient management and long-term maintenance. These include remote monitoring systems, wearable devices, and mobile applications capable of tracking patient adherence and behavioral changes over time. Conversational agents, chatbots, and mobile-based digital assistants have been designed to support patient communication, motivation, and adherence monitoring. These systems can deliver personalized oral-hygiene instructions, provide behavioral feedback, and facilitate remote follow-up through automated reminders and data tracking (16).

From a therapeutic perspective, AI-assisted decision support systems can also help clinicians simulate alternative treatment scenarios and anticipate potential responses to non-surgical or supportive periodontal therapy. However, current evidence remains largely based on retrospective analyses and proof-of-concept studies, with limited prospective validation. It is important to note that no AI system has currently been validated to autonomously recommend periodontal treatment modalities, reinforcing the role of AI as an adjunct rather than a substitute for clinical judgment (10).

Collectively, these digital solutions allow for continuous patient engagement and may support preventive strategies to reduce disease recurrence. Although early evidence suggests that these technologies may enhance patient engagement and adherence, their impact on hard clinical endpoints, such as periodontal stability and tooth retention, remains insufficiently validated. Consequently, further prospective studies are required to determine the long-term clinical benefits and cost-effectiveness of AI-assisted therapeutic and prognostic tools in periodontal care.

A concise overview of current artificial intelligence applications in periodontal diagnostics, prognosis, and treatment support, together with their level of evidence and degree of clinical validation, is summarized in Table 1.

**Table 1:** Overview of current artificial intelligence applications in periodontology, summarizing their clinical purpose, level of evidence, degree of validation, and clinical readiness.

AI application	Data source	Main purpose	Level of evidence	Clinical readiness	Key limitations
Detection of periodontal bone loss	Panoramic and periapical radiographs	Automated detection and staging of periodontitis	Systematic reviews, retrospective studies	Moderate	Limited external validation; dataset heterogeneity
Photo-based screening	Intraoral photographs	Population-level screening and tele-dentistry	Narrative and systematic reviews	Low–moderate	Image quality variability; lack of standardized protocols
Risk prediction models	Clinical and demographic data	Prediction of disease progression and treatment response	Retrospective cohort studies	Low	Limited longitudinal data; poor generalizability
Treatment outcome prediction	Longitudinal clinical datasets	Forecasting response to non-surgical therapy	Pilot ML studies	Low	Small sample sizes; lack of prospective validation
Patient engagement tools	Behavioral and self-reported data	Improving adherence and maintenance	Narrative reviews	Exploratory	Limited evidence of clinical impact

Clinical readiness was qualitatively evaluated based on study design, external or multicenter validation, sample size, and feasibility of integration into routine periodontal practice. This assessment reflects the translational maturity of each AI application, rather than a formal grading system, and is intended to help clinicians and researchers interpret the current state of evidence in a practical context.

### 2.5 Clinical benefits

The integration of artificial intelligence into periodontal practice offers potential benefits that extend beyond improvements in diagnostic accuracy alone. One of the most frequently reported advantages relates to workflow optimization and clinical efficiency. Automated image analysis, structured report generation, and data management systems can reduce the time spent on repetitive documentation tasks, thereby allowing clinicians to dedicate greater attention to patient care and complex clinical decision-making (16). In addition, AI-based platforms may contribute to greater standardization and consistency in periodontal assessment, particularly in multiclinician or multi-center settings. By supporting uniform interpretation of radiographic findings and structured periodontal charting, these systems may reduce inter-examiner variability and improve longitudinal follow-up assessments (5).

AI also plays an emerging role in treatment planning and outcome prediction. Predictive algorithms trained on longitudinal datasets can estimate the probability of treatment success, recurrence, or disease progression based on patient-specific variables, including systemic health status, smoking habits, baseline periodontal severity, and response to previous therapy (15). Such risk-based insights may help clinicians tailor therapeutic strategies and maintenance schedules to individual patient profiles, aligning with the principles of precision periodontology.

Another growing dimension of AI's clinical value involves patient engagement and adherence to periodontal care. Digital tools such as intelligent chatbots and mobile health applications have been developed to support personalized oral hygiene instructions, behavioral feedback, and appointment reminders, fostering continuous communication between patients and care providers outside the clinical setting (17). Although these approaches show promise in improving self-care behaviors, their long-term impact on periodontal stability and hard clinical outcomes remains to be fully established.

Finally, AI systems hold potential for population-level health monitoring and the development of tele-periodontology models. Remote image-based screening and automated risk stratification may facilitate earlier detection of periodontal disease and expand access to care in underserved or resource-limited settings (18).

As these technologies continue to evolve, they are expected to contribute to a more proactive, data-supported, and patient-centered approach to periodontal care delivery, provided that appropriate validation and governance frameworks are established.

### 2.6 Cost and infrastructure considerations

The implementation of AI in periodontal practice requires investment in both hardware and software, including digital imaging systems, data storage, and secure IT infrastructure. Licensing fees for AI platforms and training for clinicians may also contribute to initial costs. While these investments can be substantial, AI has the potential to improve efficiency, reduce diagnostic errors, and support preventive strategies, which may translate into long-term cost saving (16).

However, evidence on the cost-effectiveness of AI in periodontology is currently limited, and prospective studies are needed to assess whether these tools provide measurable economic benefits in routine clinical practice. Consideration of infrastructure and financial feasibility is therefore crucial when planning AI integration into dental workflows (18).

### 2.7 Clinical education and calibration

Successful implementation of AI tools in periodontology requires adequate clinician training and calibration. Clinicians must be able to correctly interpret AI outputs, integrate them into clinical decision-making, and ensure consistent application across different operators. Standardized educational programs, workshops, and inclusion of AI continuing professional development can help achieve these goals. Addressing these educational needs is crucial to maximize clinical utility, reduce variability in treatment outcomes, and promote safe and effective adoption of AI-assisted periodontal care (19).

## 3 Challenges and limitations

Although artificial intelligence offers considerable potential to enhance periodontal diagnosis and treatment, its clinical implementation is constrained by several critical challenges. These include heterogeneous data quality, limited external validation of predictive models, ethical and privacy concerns, and practical barriers to integration into routine clinical workflows. Careful consideration of these factors is therefore essential to enable the safe, reliable, and effective translation of AI technologies into periodontal practice (7).

### 3.1 Methodological and data-related limitations

Despite the growing interest in artificial intelligence applications in periodontology, several methodological limitations currently hinder their translation into routine clinical practice. Among these, issues related to data quality, standardisation, and model validation constitute the most substantial barriers to the development of robust,eneralizable AI-based systems (8).

Most AI models in periodontology are trained on retrospective datasets characterized by substantial heterogeneity in imaging modalities, acquisition protocols, clinical assessment methods, and patient populations. Variability in radiographic quality, device-specific characteristics, and annotation strategies can significantly influence model performance and limit reproducibility across different clinical settings (12). Additionally, differences in periodontal case definitions and diagnostic criteria across studies may substantially influence the training, labeling, and performance of artificial intelligence models. Variability in the use of clinical attachment level thresholds, probing depth cut-offs, radiographic bone loss criteria, and disease staging systems can introduce inconsistencies in ground truth annotation, thereby affecting model learning and output reliability. Such heterogeneity may limit the comparability of results across studies and reduce the external validity and generalizability of AI-based models when applied to different clinical settings or populations. Consequently, the adoption of standardized periodontal case definitions and diagnostic frameworks is crucial to ensure robust model development, transparent validation, and meaningful comparison of AI performance across studies (10).

Periodontal clinical parameters such as probing depth and clinical attachment level are inherently operator-dependent, introducing noise and bias into machine learning models when inconsistently recorded (5). Model validation represents a further critical limitation. Many published studies rely on small, single-center datasets and internal validation strategies, frequently using image-level rather than patient-level data splits. This practice may artificially inflate performance metrics and does not adequately reflect real-world clinical scenarios, where patient heterogeneity and temporal disease progression are key determinants of treatment outcomes (9). Furthermore, high diagnostic accuracy does not necessarily equate to clinical usefulness, particularly when outcome definitions are inconsistent or insufficiently aligned with established periodontal diagnostic and prognostic frameworks. In addition to these methodological constraints, the clinical adoption of AI-based systems in periodontology raises important ethical, regulatory, and governance-related considerations. The limited interpretability of many deep learning models may hinder clinician trust and complicate accountability in clinical decision-making. Moreover, unresolved issues related to data privacy, regulatory approval, and medico-legal responsibility persist, particularly in the absence of shared guidelines for the deployment of AI tools in periodontal care. These challenges underscore the need for AI technologies to be implemented as transparent, clinician-supervised decision-support systems rather than as autonomous diagnostic or therapeutic solutions (7).

### 3.2 Ethical, interpretability, and clinical integration challenges

Beyond methodological limitations, the clinical adoption of artificial intelligence in periodontology is further challenged by ethical, interpretability, and implementation-related issues. Many AI systems, particularly deep learning–based models, operate as so-called “black boxes,” generating predictions without providing transparent or easily interpretable explanations. Limited model interpretability has been widely recognized as a major barrier to clinical trust, accountability, and safe deployment of AI systems in healthcare, especially in diagnostic and decision-support contexts where clinicians remain legally and ethically responsible for patient care (20) considerations are closely linked to data governance and patient privacy. AI applications in periodontology often rely on large volumes of clinical and imaging data, raising concerns related to informed consent, data ownership, algorithmic bias, and compliance with data protection regulations such as the General Data Protection Regulation (GDPR). Inadequate governance frameworks may increase the risk of biased model behavior and inequitable clinical performance, particularly when training datasets are not representative of diverse patient populations. From a practical standpoint, the integration of AI-based tools into routine periodontal workflows remains limited. Barriers include lack of interoperability with existing clinical software, costs associated with implementation and maintenance, and limited training of dental professionals in AI-assisted decision-making. In the absence of shared clinical guidelines and regulatory standards specific to AI use in dentistry, these factors continue to restrict widespread clinical adoption, reinforcing the need for AI systems to function as transparent, clinician-supervised decision-support tools rather than autonomous diagnostic or therapeutic solutions.

Moreover,the adoption of AI tools is increasingly influenced by emerging regulatory frameworks. Regulatory authorities such as the U.S. Food and Drug Administration (FDA) and European regulators are actively developing guidelines for medical devices based on AI and machine learning, with a focus on safety, transparency, continuous performance monitoring, and risk management. At the same time, compliance with data protection regulations, including the General Data Protection Regulation (GDPR), is a fundamental requirement for the clinical implementation of AI systems. Understanding and adhering to these constantly evolving regulatory frameworks is essential to ensure patient safety, ethical implementation, and legal accountability when integrating AI tools into periodontal clinical practice (21).

## 4 Future perspectives and conclusions

The rapid development of artificial intelligence technologies has opened new opportunities for detecting periodontal disease, assessing risk, and managing patients. Future research efforts should prioritise the generation of high-quality, multicenter datasets and the adoption of standardised reporting and validation frameworks to improve the robustness, transparency, and generalizability of AI-based models. Prospective and longitudinal studies, including AI-assisted clinical trials, will be essential to determine whether these tools can meaningfully improve clinical outcomes beyond experimental settings. The integration of AI into periodontology should be guided by the concept of augmented, rather than autonomous, intelligence, where AI systems support clinicians by enhancing diagnostic consistency, risk stratification, and treatment planning while preserving professional judgment and accountability. Advances in explainable artificial intelligence and clearer regulatory pathways will be crucial to foster clinician trust and ensure safe implementation in routine practice.

In conclusion, artificial intelligence holds considerable promise for advancing periodontal care, but its current applications remain largely investigational. At present, AI-based tools should be regarded as decision-support systems rather than replacements for clinical expertise. Continued interdisciplinary collaboration among clinicians, data scientists, and regulatory bodies will be essential to translating AI innovations into clinically reliable, ethically sound, and patient-centred solutions in periodontology.
